# Non-contiguous finished genome sequence and description of *Oceanobacillus massiliensis* sp. nov.

**DOI:** 10.4056/sigs.4267953

**Published:** 2013-12-15

**Authors:** Véronique Roux, Matthieu Million, Catherine Robert, Alix Magne, Didier Raoult

**Affiliations:** 1 Aix Marseille Université, URMITE, Faculté de médecine, Aix-Marseille Université, Marseille, France

**Keywords:** *Oceanobacillus massiliensis*, *Firmicutes*, Polar flagellum

## Abstract

*Oceanobacillus massiliensis* strain N’Diop^T^ sp. nov. is the type strain of *O. massiliensis* sp. nov., a new species within the genus *Oceanobacillus*. This strain, whose genome is described here, was isolated from the fecal flora of a healthy patient. *O. massiliensis* is an aerobic rod. Here we describe the features of this organism, together with the complete genome sequence and annotation. The 3,532,675 bp long genome contains 3,519 protein-coding genes and 72 RNA genes, including between 6 and 8 rRNA operons.

## Introduction

*Oceanobacillus massiliensis* strain N’diop^T^ (CSUR 132^T^ = DSM 24644 ^T^) is the type strain of *O. massiliensis* sp. nov. This bacterium is a Gram-positive strictly aerobic rod, motile by a polar flagellum and was isolated from the stool of a healthy Senegalese patient as part of a "culturomics" study aiming at cultivating individually all species within human feces [1].

Presently, "the gold standard method" to define a bacterial species is DNA-DNA hybridization [2]. But this method is time-consuming and the inter-laboratory reproducibility is poor. So, with the development of PCR and sequencing methods, 16S rRNA gene sequence comparison with internationally-validated cutoff values constitutes, for most bacterial genera, a reliable, reproducible and comparable tool that enables the taxonomic classification of new bacterial species [3]. Recently, it was proposed that new bacterial taxa be described using a polyphasic approach [4] that would include the genome sequence, MALDI-TOF spectrum and main phenotypic characteristics (habitat, Gram-stain reaction, culture, cell wall structure and metabolic characteristics).

Here we present a summary classification and a set of features for *O. massiliensis* sp. nov. strain Ndiop^T^ together with the description of the complete genomic sequencing and annotation. These characteristics support the circumscription of the species *O. massiliensis*.

The genus *Oceanobacillus* was first described by Lu *et al.* [5] and was emended by Yumoto *et al.* [6]. The genus comprises 12 recognized species and two subspecies. These bacteria are motile Gram-positive rods, obligately aerobic or facultatively anaerobic, obligately or facultatively alkaliphilic. These species were isolated from deep-sea sediment core [5,7], deteriorated mural paintings [8], salt field [9], freshwater fish [10], algal mat [11], insects in freshwater [12], *Bacillus*-dominated wastewater treatment system in Korea [13], fermented shrimp paste samples [14], soy sauce production equipment [15], a marine solar saltern [16], activated sludge in a bioreactor [17], traditional Korean fermented food [18] and a fermented Polygonum indigo liquor sample [19]. These bacteria belong to the phylum *Firmicutes*, in the family *Bacillaceae*. There is no evidence of pathogenicity of these bacteria.

## Classification and features

A stool sample was collected from a healthy 16-year-old male Senegalese volunteer patient living in N’diop (a rural village in the Guinean-Sudanian zone in Senegal), who was included in a research protocol. The patient gave an informed and signed consent, and the agreements of the National Ethics Committee of Senegal and the local ethics committee of the IFR48 (Marseille, France) were obtained under agreement 09-022 and 11-017. The fecal specimen was preserved at -80°C after collection and sent to Marseille. Strain N’diop (Table 1) was isolated in January 2011 by aerobic cultivation on 5% sheep blood-enriched Columbia agar (BioMerieux).

**Table 1:** Classification and general features of *Oceanobacillus massiliensis* strain N’Diop^T^ according to the MIGS recommendations [20]

**MIGS ID**	**Property**	**Term**	**Evidence code^a^**
	Current classification	Domain *Bacteria*	TAS [21]
		Phylum *Firmicutes*	TAS [22-24]
		Class *Bacilli*	TAS [25,26]
		Order *Bacillales*	TAS [27,28]
		Family *Bacillaceae*	TAS [28,29]
		Genus *Oceanobacillus*	TAS [5,30-33]
		Species *Oceanobacillus massiliensis*	IDA
		Type strain N’Diop^T^	IDA
	Gram stain	Positive	IDA
	Cell shape	Bacilli	IDA
	Motility	Motile by polar flagellum	IDA
	Sporulation	Nonsporulating	IDA
	Temperature range	Mesophile	IDA
	Optimum temperature	37°C	IDA
MIGS-6.3	Salinity	Growth in BHI medium + 5% NaCl	IDA
MIGS-22	Oxygen requirement	Aerobic	IDA
	Carbon source	Unknown	NAS
	Energy source	Unknown	NAS
MIGS-6	Habitat	Human gut	IDA
MIGS-15	Biotic relationship	Free living	IDA
MIGS-14	Pathogenicity Biosafety level Isolation	Unknown 2 Human feces	NAS
MIGS-4	Geographic location	Senegal	IDA
MIGS-5	Sample collection time	September 2009	IDA
MIGS-4.1	Latitude	13.41	IDA
MIGS-4.1	Longitude	-16.22	IDA
MIGS-4.3	Depth	Surface	IDA
MIGS-4.4	Altitude	<100 m above sea level	IDA

Evidence codes - IDA: Inferred from Direct Assay; TAS: Traceable Author Statement (i.e., a direct report exists in the literature); NAS: Non-traceable Author Statement (i.e., not directly observed for the living, isolated sample, but based on a generally accepted property for the species, or anecdotal evidence). These evidence codes are from the Gene Ontology project [34]. If the evidence is IDA, then the property was directly observed for a live isolate by one of the authors or an expert mentioned in the acknowledgements.

The bacterial DNA was extracted using the MagNA Pure LC DNA isolation kit III (Roche, Mannheim, Germany) with the MagNA Pure LC instrument as described by the manufacturer.

**Figure 1:**
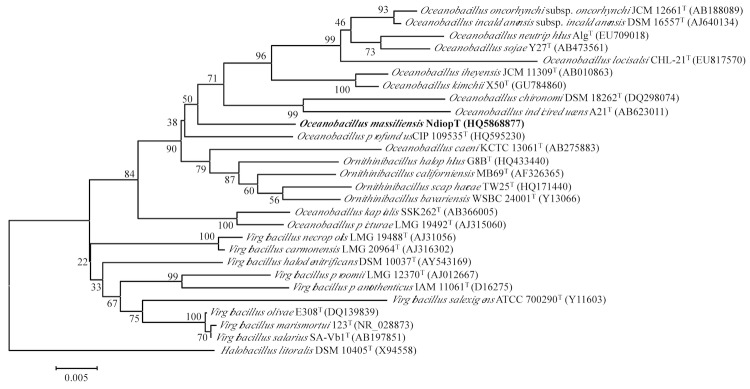
Phylogenetic tree highlighting the position of *Oceanobacillus massiliensis* strain N’Diop^T^ relative to other type strains within the *Oceanobacillus*, *Ornithinibacillus*, and *Virgibacillus* genera. GenBank accession numbers are indicated in parentheses. Sequences were aligned using CLUSTALX, and phylogenetic inferences obtained using the neighbor joining method within the MEGA 5 software [18]. Numbers at the nodes are percentages of bootstrap values obtained by repeating the analysis 1,000 times to generate a majority consensus tree. *Halobacillus litoralis* was used as outgroup. The scale bar represents 0.005 nucleotide change per nucleotide position.

PCR amplification of the 16S rRNA gene was performed using the universal primer pair fD1 and rp2 [35]. PCR products were purified using MultiScreen PCR (Millipore) and sequencing reactions were carried out using a DNA sequencing kit (BigDye Terminator Cycle Sequencing v1.1 Ready Reactions, PE biosystems) according to the manufacturer’s instructions. Sequencing products were purified and electrophoresis was performed with the 3130 Genetic Analyzer (Applied Biosystems). Base insertions were noted at the beginning and the end of the gene sequence. So, we tried to clone PCR products in pGEM-T Easy Vector (Promega) as described by the manufacturer. But the sequence was too long and no result was obtained. A new primer Omassr (GCCTGCAATCCGAACTGAGA) was chosen to reduce the length of fragment when combined with fD1 to perform PCR amplification. Seventeen clones were PCR amplified using the primers M13d (CGCCAGGGTTTTCCCAGTCACGAC) and M13r (TCACACAGGAAACAGCTATGAC) and the obtained products were sequenced. The obtained sequences were compared with sequences deposited in the GenBank database by using the BLAST program through the NCBI server. This strain exhibited a 96.4-97% nucleotide sequence similarity with *O. profundus*, the phylogenetically closest validated *Oceanobacillus* species. Gene sequences were aligned using the multisequence alignment program CLUSTAL X (1.8) [36]. Phylogenetic relationships with closely related species were determined by using MEGA version 5.0 [37]. Distance matrices were determined following the assumptions described by Kimura and were used to elaborate a dendrogram using the neighbor-joining method (Figure 1). The maximum-parsimony algorithm was also used to infer phylogenetic analysis. A bootstrap analysis (bootstrap values were obtained for a consensus tree based on 1,000 randomly generated trees) was performed to investigate the stability of the trees obtained. The clustering of the new isolate was the same with the two methods.

For DNA-DNA hybridization, cells were disrupted by using a French pressure cell (Thermo Spectronic) and the DNA in the crude lysate was purified by chromatography on hydroxyapatite as described by Cashion *et al.* [38]. DNA-DNA hybridization was carried out as described by De Ley *et al.* [39] under consideration of the modifications described by Huss *et al.* [40] using a model Cary 100 Bio UV/VIS-spectrophotometer equipped with a Peltier-thermostatted 6x6 multicell changer and temperature controller with in-situ temperature probe (Varian). DNA-DNA reassociation rate was 25.5% between strain Ndiop^T^ and *Oceanobacillus profundus*. So, they did not belong to the same species when the recommendations of a threshold value of 70% DNA-DNA similarity for the definition of bacterial species by the *ad hoc* committee are considered [41].

**Figure 2:**
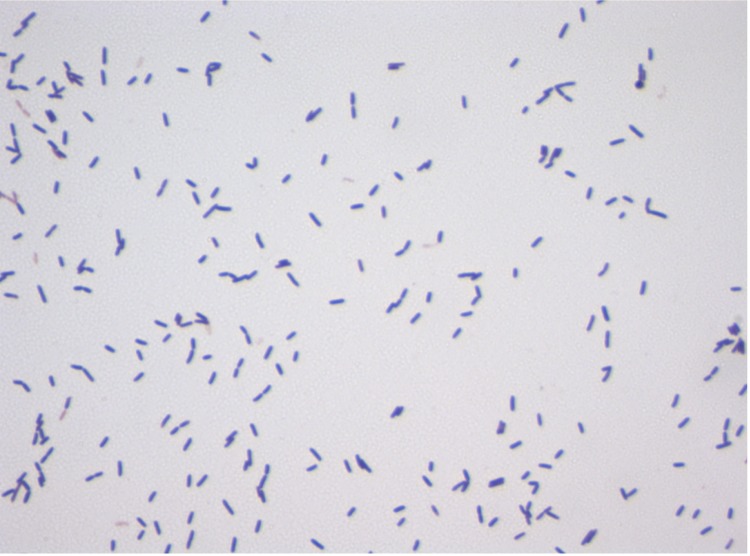
Gram staining of *O. massiliensis* strain N’Diop^T^

Surface colonies were observed on sheep blood agar (bioMérieux) after 24 h aerobic incubation at 37°C. The colonies of the strain N’diop^T^ were circular, greyish, shiny and smooth, 2-5 mm in diameter. Gram staining showed Gram-positive bacilli (Figure 2). Different growth temperatures (25, 30, 37, 45 and 50°C) were tested. Growth occurred between 25°C and 45°C, and optimal growth was observed between 30°C and 37°C. Growth of the strain was tested under aerobic atmosphere, in the presence of 5% CO_2_, and also in anaerobic and microaerophilic atmospheres which were created using GENbag anaer and GENbag microaer (bioMérieux), respectively. The strain was aerobic and also grew in microaerophilia and in the presence of 5% CO_2_ but did not grow in an anaerobic atmosphere. The NaCl concentrations allowing growth of strain N’Diop^T^, were determined on Difco^TM^Brain Heart Infusion Agar plates (Becton Dickinson). The powder was supplemented with NaCl (Euromedex) to obtain the tested concentrations (0.5, 1, 2, 3, 5 10, 15%, w/v). Growth occurred between 0.5-10% NaCl but the optimum growth was between 0.5-5% NaCl. Growth in the range of pH 5.0-10.0 was tested using BBL^TM^ Brain Heart Infusion (Becton Dickinson) supplemented with 5% NaCl. The final pH was adjusted with HCl or NaOH solution. Growth occurred between pH 7-9 but optimum pH was 7-8.

**Figure 3:**
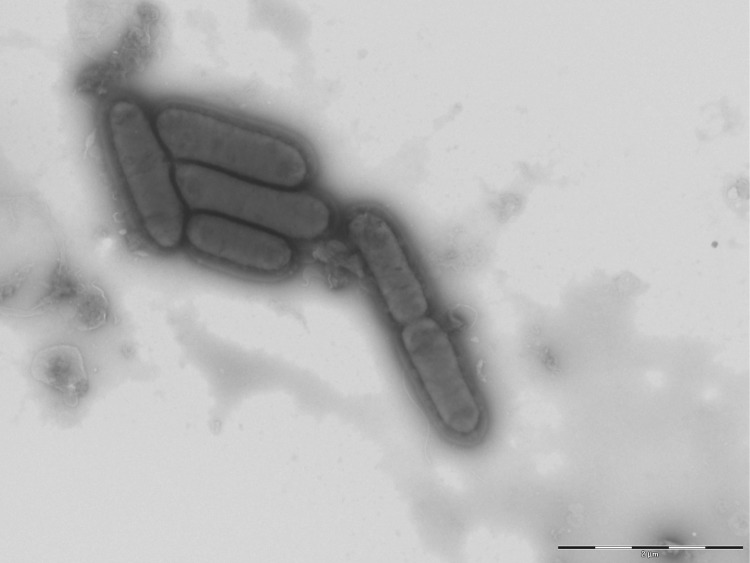
Transmission electron microscopy of *O. massiliensis* strain N’Diop^T^, using a Morgani 268D (Philips) at an operating voltage of 60kV. The scale bar represents 1 μm.

The size and ultrastructure of cells were determined by negative staining transmission electron microscopy. Cells were grown on Columbia agar with 5% sheep blood for 24 h at 37°C. The bacteria were suspended in phosphate buffer (Gibco; KCl 200 mg/L, KH_2_PO_4_ 200 mg/L, NaCl 8 g/L, Na_2_HPO_4_-7H_2_O 2.16 g/L) and pre-fixed in 5% (v/v) glutaraldehyde in phosphate buffer for at least 1 h at room temperature, washed in the same buffer and stained with 1% (w/v) phosphotungstic acid. The samples were examined on a Morgagni 268D (Philips) electron microscope at an operating voltage of 60 kV. The rods were 1.2-1.9 μm long and 0.4-0.7 μm wide (Figure 3). Polar flagella were observed.

Strain N’Diop^T^ exhibited catalase activity and oxidase activity. The commercially available Api ZYM, Api 20NE (bioMérieux) were used to characterize the biochemical properties of the strain according to the manufacturer’s instructions and incubation was performed at 37 °C for 4h and 24h, respectively. Api 50 CH strips were inoculated with a heavy bacterial suspension in Api 50CHB/E medium supplemented with 5% NaCl (w/v) and incubation was performed at 37°C for 48h. Phenotypic characteristics were compared to those of species that were most closely related in terms of 16S rRNA gene sequences. Characteristic traits are presented in Table 2.

**Table 2:** Diagnostic trait differentiation.

Characteristic	1	2	3	4	5	6	7	8	9	10
Assimilation of										
Glycerol	w	w	+	+	w	-	w	-	-	-
L-Arabinose	-	-	-	+	-	-	w	-	-	-
Ribose	-	+	-	+	-	-	-	-	-	-
D-Xylose	+	+	-	+	-	-	w	-	-	-
Methyl-βD-Xylopyranoside	-	-	-	+	-	-	-	-	-	-
D-Galactose	-	-	+	+	-	-	-	-	-	-
D-Glucose	+	+	+	+	w	-	w	-	-	-
D-Fructose	+	+	+	+	w	-	w	-	-	-
D-Mannose	+	+	+	+	w	-	w	-	-	-
L-Sorbose	-	-	+	-	-	-	-	-	-	-
Dulcitol	-	-	+	+	-	-	-	-	-	-
Inositol	-	-	+	+	-	-	-	-	-	-
D-Mannitol	+	+	+	+	-	-	-	-	-	-
Methyl-αD-Mannopyranoside	-	-	-	-	-	-	w	-	-	-
Methyl-αD-Glucopyranoside	-	-	+	+	-	-	w	-	-	-
N-Acetylglucosamine	+	+	+	+	w	-	w	-	-	-
Amygdalin	-	+	+	+	-	-	-	-	-	-
Arbutin	-	-	+	+	-	-	-	-	-	-
Salicin	+	+	+	+	-	-	w	-	-	-
D-Cellobiose	+	+	+	+	-	-	w	-	-	-
D-Maltose	+	+	+	+	-	-	w	-	-	-
D-Saccharose	-	+	+	+	-	-	-	-	-	-
D-Trehalose	+	+	+	+	-	-	-	-	-	-
D-Raffinose	-	-	+	+	-	-	-	-	-	-
Gentiobiose	-	-	+	+	-	-	-	-	-	-
D-Turanose	-	-	+	+	-	-	-	-	-	-
D-Tagatose	-	+	+	+	-	-	-	-	-	-
D-Arabitol	-	w	w	w	-	-	-	-	-	-
potassium Gluconate	-	-	w	w	-	+	-	-	-	-
Reduction of nitrates to nitrites	-	+	+	+	-					
Urease	-	+	-	-	-	-	-	-	-	-
Esculin hydrolysis	+	+	+	+	-	+	-	-	-	-
Para-nitrophenyl-βD-galactopyranosidase	-	w	-	w	-	+	w	-	+	+
Esterase (C4)	+	+	+	+	+	+	+	-	-	-
Esterase lipase (C8)	+	+	+	+	w	+	w	w	w	+
Leucine arylaminidase	-	-	-	-	w	w	-	+	w	-
α-chemotrypsin	-	-	w	-	-	-	w	-	-	-
β-glucuronidase	-	+	-	-	-	-	-	+	+	-
α-glucosidase	+	-	+	-	-		-	-	-	-

Strains: 1, *O. massiliensis* sp. nov. Ndiop^T^; 2, *O. profundus* CIP 109535^T^ ; 3, *O. oncorhynchi subsp. oncorhynchi* CIP 108867^T^; 4, *O. oncorhynchi subsp. incaldanensis* CIP 109235^T^; 5, *O. iheyensis* CIP 107618^T^; 6, *O. chironomi* CIP109536^T^ ; 7, *O. picturae* CIP 108264^T^; 8, *Ornithinibacillus bavariensis* DSM 15681^T^; 9, *Ornithinibacillus californiensis* DSM 16628^T^; 10, *Ornithinibacillus contaminans* DSM 22953^T^.

+: positive result, -: negative result, w: weak positive result

Analysis of respiratory quinones by HPLC was carried out by the Identification Service and Dr Brian Tindall, DSMZ, Braunschweig, Germany. Respiratory lipoquinones were extracted from 100 mg of freeze dried cell material as described by Tindall [42,43]. Respiratory lipoquinones were separated into their different classes (menaquinones and ubiquinones) by thin layer chromatography on silica gel, using hexane:*ter*-butylmethylether (9:1 v/v) as solvent. UV absorbing bands corresponding to menaquinones or ubiquinones were removed from the plate and further analyzed by HPLC at 269 nm. The respiratory quinones were MK-7 (97%) and MK6 (7%) for strain N’Diop^T^. Preparation and determination of cellular fatty acids were carried out by following the procedures given for the Sherlock Microbial identification System (MIDI). The major fatty acids were C15:0 anteiso 63.24% and C17:0 anteiso 26.86%. The DNA base composition was determined by using the HPLC method of Mesbah *et al.* [44]. The value found for strain N’diop ^T^ was 40.8%. Polar lipids were extracted from 100 mg of freeze dried cell material using a chloroform:methanol:0.3% aqueous NaCl mixture 1:2:0.8 (v/v/v) (modified after [45]). The extraction solvent was stirred overnight and the cell debris pelleted by centrifugation. Polar lipids were recovered into the chloroform phase by adjusting the chloroform:methanol:0.3% aqueous NaCl mixture to a ratio of 1:1:0.9 (v/v/v). Polar lipids were separated as previously described [46]. The polar lipids present were diphosphatidylglycerol, phosphatidylglycerol, phospholipid 1, an unidentified aminolipid and sulfoquinovosyl diacylglycerol. The whole cells of *O. massiliensis* were hydrolyzed (4N HCl, 100 °C, 16 hours) and the hydrolysates were subjected to thin-layer chromatography on cellulose plates using the solvent system of Rhuland *et al.* [47]. *Meso*-diaminopimelic acid (*meso*-Dpm) was found as diagnostic diamino acid of the peptidoglycan. The occurrence of meso-Dpm has been reported up to now only for the peptidoglycan type A1γ (and A1γ’ with glycine instead of L-alanine) and for three variations of peptidoglycan type A4γ (A31.1, A31.2 and A31.3, see [48]). The variations of peptidoglycan type A4γ based on meso-Dpm have been found so far exclusively in members of the genera *Brachybacterium*, *Devriesea* and *Dermabacter* (family *Dermabacteraceae*). Because a close relationship between *O. massiliensis* and the family *Dermabacteraceae* can be ruled out, we conclude the peptidoglycan type to be A1γ.

Antimicrobial susceptibility was determined according to the National Committee for Clinical Laboratory Standards (NCCLS) criteria. Strain N’diop^T^ was found to be susceptible to doxycycline, rifampicine, vancomycine, nitrofurantoin, amoxicillin, erythromycin, ampicillin, ceftriaxone, ciprofloxacine, gentamycine, penicilline, imipenem. But it was resistant to trimethoprim/sulfamethoxazole and metronidazole.

**Figure 4:**
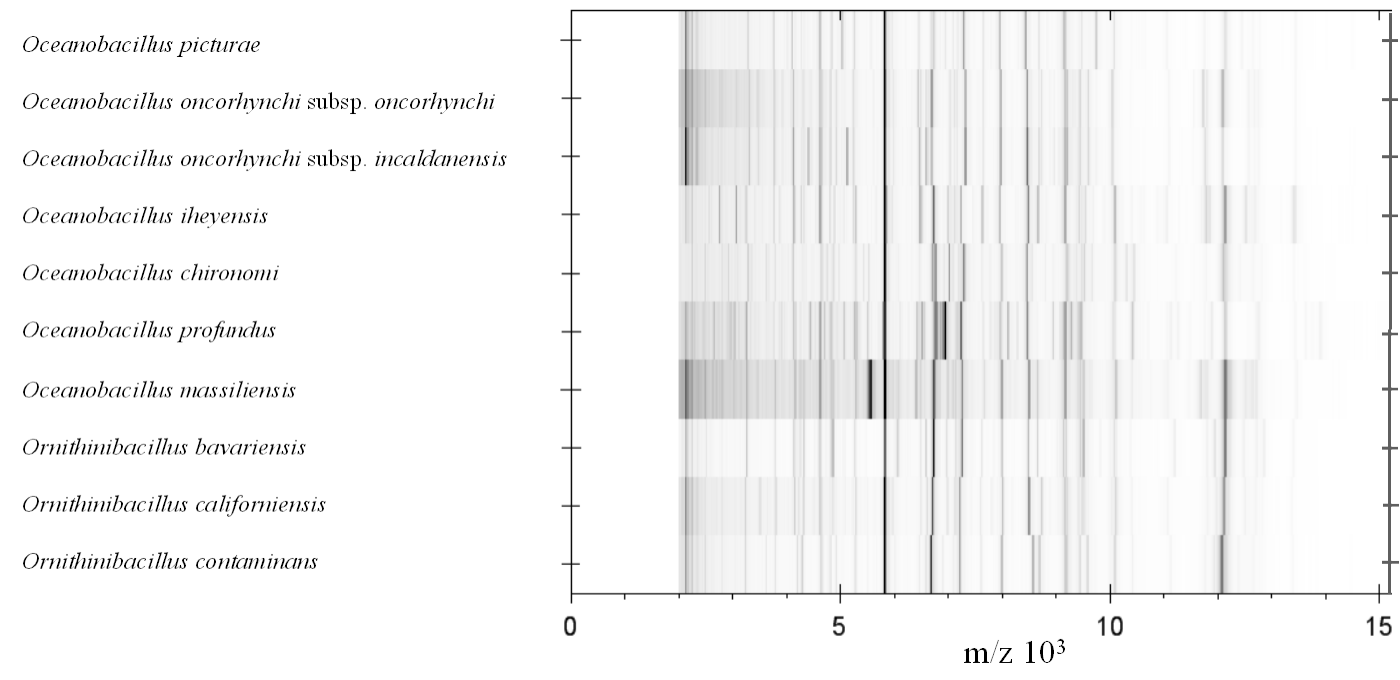
Reference mass spectra from *O. massiliensis* strain N’Diop^T^, other representatives of the genus *Oceanobacillus* and type strains of the genus *Ornithinibacillus*. Spectra from 24 individual colonies were compared and a reference spectrum was generated.

Matrix-assisted laser-desorption/ionization time-of-flight (MALDI-TOF) MS protein analysis was carried out. Briefly, a pipette tip was used to pick one isolated bacterial colony from a culture agar plate, and to spread it as a thin film on a MALDI-TOF target plate (Bruker Daltonics). Twelve distinct deposits were done for strain N’Diop^T^ from twelve isolated colonies and the manipulation was repeated another day. After air-drying, 1.5 µl matrix solution (saturated solution of α-cyanohydroxycinnaminic acid in 50% aqueous acetonitrile containing 2.5% trifluoroacetic acid) per spot was applied. MALDI-TOF MS was conducted using the Microflex LT spectrometer (Bruker Daltonics). All spectra were recorded in linear, positive ion mode. The acceleration voltage was 20 kV. Spectra were collected as a sum of 240 shots across a spot. Preprocessing and identification steps were performed using the manufacturer’s parameters. The N’Diop^T^ spectra were imported into the MALDI BioTyper software (version 3.0, Bruker) and analyzed by standard pattern matching (with default parameter settings) against the main spectra of 4,108 bacteria including the spectra from *Oceanobacillus profundus* CIP 109535^T^, *Oceanobacillus picturae* CIP 108264^T^, *Oceanobacillus chironomi* CIP 109536^T^, *Oceanobacillus iheyensis* CIP 107618^T^, *Oceanobacillus oncorhynchi subsp. oncorhynchi* CIP 108867^T^ and *Oceanobacillus oncorhynchi subsp incaldanensis* CIP 109235^T^ which were the most closely related species when 16S rRNA gene sequences were compared and *Ornithinibacillus bavariensis* DSM 15681^T^, *Ornithinibacillus californiensis* DSM 16628^T^ and *Ornithinibacillus contaminans* DSM 22953^T^, used as reference data, in the BioTyper database. A score enabled the identification, or not, from the tested species: a score > 2.3 with a validated species enabled the identification at the species level, a score > 1.7 but < 2 enabled the identification at the genus level; and a score < 1.7 did not enable any identification. For strain N’Diop^T^, none of the obtained scores were > 1.5, thus suggesting that our isolate was not a member of a known species. We incremented our database with the spectrum from strain N’Diop^T^ (Figure 4). The spectrum was made available online in our free-access URMS database [49].

## Genome sequencing information

### Genome project history

The organism was selected for sequencing on the basis of its phylogenetic position and 16S rRNA similarity to other members of the genus *Oceanobacillus*, and is part of a “culturomics” study of the human digestive flora aiming at isolating all bacterial species within human feces. It was the second sequenced genome of an *Oceanobacillus* species, *Oceanobacillus massiliensis* sp. nov. A summary of the project information is shown in Table 3. The EMBL accession number is CAER01000000 and consists of 95 contigs (≥500 bp) and 9 scaffold (> 2,500 bp). Table 2 shows the project information and its association with MIGS version 2.0 compliance.

**Table 3:** Project information

**MIGS ID**	**Property**	**Term**
MIGS-31	Finishing quality	High-quality draft
MIGS-28	Libraries used	One paired end 3-kb library and one Shotgun library
MIGS-29	Sequencing platforms	454 GS FLX Titanium
MIGS-31.2	Fold coverage	23x
MIGS-30	Assemblers	Newbler version 2.5.3
MIGS-32	Gene calling method	Prodigal
	EMBL ID	CAER01000000
	EMBL Date of Release	February 28,2013
	Project relevance	Study of the human gut microbiome

### Growth conditions and DNA isolation

*O. massiliensis* sp. nov. strain N’Diop^T^, CSUR P132^T^, DSM 24644^T^, was grown aerobically on 5% sheep blood-enriched Columbia agar at 37°C. Three petri dishes were spread and resuspended in 3x100µl of G2 buffer. A first mechanical lysis was performed by glass powder on the Fastprep-24 device (Sample Preparation system) from MP Biomedicals, USA during 2x20 seconds. DNA was then incubated for a lysozyme treatment (30 minutes at 37°C) and extracted through the BioRobot EZ 1 Advanced XL (Qiagen). The DNA was then concentrated and purified on a Qiamp kit (Qiagen). The yield and the concentration were measured by the Quant-it Picogreen kit (Invitrogen) on the Genios Tecan fluorometer at 139 ng/µl.

### Genome sequencing and assembly

Shotgun and 3-kb paired-end sequencing strategies were performed. The shotgun library was constructed with 500 ng of DNA with the GS Rapid library Prep kit (Roche). For the paired-end sequencing, 5 µg of DNA was mechanically fragmented on a Hydroshear device (Digilab) with an enrichment size at 3-4 kb. The DNA fragmentation was visualized using the 2100 BioAnalyzer (Agilent) on a DNA labchip 7500 with an optimal size of 3.1 kb. The library was constructed according to the 454 GS FLX Titanium paired-end protocol. Circularization and nebulization were performed and generated a pattern with an optimal size of 450 bp. After PCR amplification through 17 cycles followed by double size selection, the single stranded paired-end library was then quantified using the Genios fluorometer (Tecan) at 220 pg/µL. The library concentration equivalence was calculated as 8.97 E+08 molecules/µL. The library was stored at -20°C until further use.

The shotgun and paired-end libraries were clonally-amplified with 3 cpb and 1 cpb in 3SV-emPCR reactions respectively on the GS Titanium SV emPCR Kit (Lib-L) v2 (Roche). The yields of the emPCR were 15.1% and 12.1% respectively. Approximately 790,000 beads for the shotgun application and for the 3kb paired end were loaded on the GS Titanium PicoTiterPlate PTP Kit 70x75 and sequenced with the GS FLX Titanium Sequencing Kit XLR70 (Roche). The run was performed overnight and then analyzed on the cluster through the gsRunBrowser and Newbler assembler (Roche). A total of 279,999 passed filter wells were obtained and generated 81 Mb with a length average of 289 bp. The passed filter sequences were assembled using Newbler with 90% identity and 40 bp as overlap. The final assembly identified 9 scaffolds and large 82 contigs (>1,500 bp).

### Genome annotation

Open Reading Frames (ORFs) were predicted using Prodigal [50] with default parameters but the predicted ORFs were excluded if they were spanning a sequencing GAP region. The predicted bacterial protein sequences were searched against the GenBank database [51] and the Clusters of Orthologous Groups (COG) databases [52] using BLASTP. The tRNAscan-SE tool [53] was used to find tRNA genes, whereas ribosomal RNAs were found by using RNAmmer [54].

Transmembrane domains and signal peptides were predicted using TMHMM [55] and SignalP [56], respectively. ORFans were identified if their BLASTp *E*-value was lower than 1e-03 for alignment length greater than 80 amino acids. If alignment lengths were smaller than 80 amino acids, we used an *E*-value of 1e-05. Such parameter thresholds have been used in previous works to define ORFans.

To estimate the mean level of nucleotide sequence similarity at the genome level between *O. massiliensis* and *O. iheyensis* (GenBank accession number PRJNA57867), the only available *Oceanobacillus* genome to date, we compared the ORFs only using comparison sequence based in the server RAST [57] at a query coverage of ≥70% and a minimum nucleotide length of 100 bp.

## Genome properties

**Figure 5:**
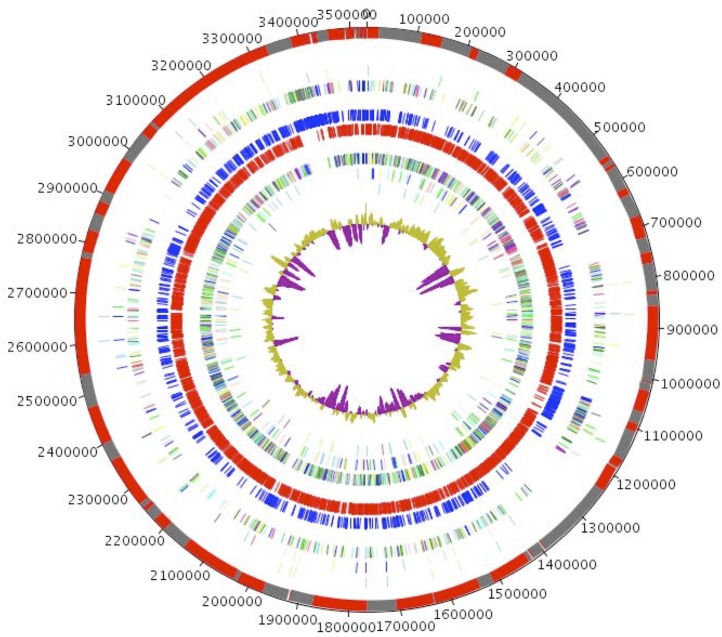
Graphical circular map of the *O. massiliensis* strain N’Diop genome. From outside to the center: Contigs (red / grey), COG category of genes on the forward strand (three circles), genes on forward strand (blue circle), genes on the reverse strand (red circle), COG category on the reverse strand (three circles), G+C content.

The genome is 3,532,675 bp long with 40.35% GC content (Table 4 and Figure 5). It is composed of 95 Contigs (9 Scaffolds). Of the 3,589 predicted genes, 3,519 were protein-coding genes, and 72 were RNAs (1 gene is 16S rRNA, 1 gene is 23S rRNA, 9 genes are 5S rRNA, and 61 are tRNA genes). A total of 2,536 genes (72.07%) were assigned a putative function (by cogs or by NR blast). In addition, 84 genes were identified as ORFans (2.39%). The remaining genes were annotated as hypothetical proteins (618 genes (17.56%)). The distribution of genes into COGs functional categories is presented in Table 5. The properties and the statistics of the genome are summarized in Tables 4 and 5. Two CRISPRs were found using CRISPERfinder program online [58] which included at least 48 predicted spacer regions (contigs 39-41) and 13 predicted spacer regions (contig 92).

**Table 4:** Nucleotide content and gene count levels of the genome

Attribute	Value	% of total^a^
Genome size (bp)	3,532,675	100
DNA coding region (bp)	2,971,565	84,12
DNA G+C content (bp)	1,425,470	40,35
Total genes	3,589	100
RNA genes	72	1.95
Protein-coding genes	3,519	98.05
Genes with function prediction	2,536	72.07
Genes assigned to COGs	2,673	75.96
Genes with peptide signals	348	9.89
Genes with transmembrane helices	885	25.15

a) The total is based on either the size of the genome in base pairs or the total number of protein coding genes in the annotated genome.

**Table 5:** Number of genes associated with the 25 general COG functional categories

**Code**	**Value**	**%age**	**Description**
J	170	4.83	Translation
A	0	0	RNA processing and modification
K	232	6.59	Transcription
L	184	5.23	Replication, recombination and repair
B	1	0.03	Chromatin structure and dynamics
D	37	1.05	Cell cycle control, mitosis and meiosis
Y	0	0	Nuclear structure
V	48	1.36	Defense mechanisms
T	142	4.04	Signal transduction mechanisms
M	156	4.43	Cell wall/membrane biogenesis
N	64	1.82	Cell motility
Z	0	0	Cytoskeleton
W	0	0	Extracellular structures
U	52	1.48	Intracellular trafficking and secretion
O	104	2.96	Posttranslational modification, protein turnover, chaperones
C	149	4.23	Energy production and conversion
G	194	5.51	Carbohydrate transport and metabolism
E	283	8.04	Amino acid transport and metabolism
F	84	2.39	Nucleotide transport and metabolism
H	100	2.84	Coenzyme transport and metabolism
I	84	2.39	Lipid transport and metabolism
P	224	6.37	Inorganic ion transport and metabolism
Q	54	1.53	Secondary metabolites biosynthesis, transport and catabolism
R	424	12.05	General function prediction only
S	281	7.99	Function unknown
X	846	24.04	Not in COGs

a) The total is based on the total number of protein coding genes in the annotated genome.

## Comparison with other *Oceanobacillus* genomes

To date, only one genome of bacteria belonging to the genus *Oceanobacillus* was sequenced, *Oceanobacillus iheyensis*. By comparison with the genome *of O. iheyensis*, *O. massiliensis* had a smaller genome (3.53 *vs* 3.63 Mb, respectively), a higher G+C content (40.35 *vs* 35.7%) but a smaller number of predicted genes (3,589 *vs* 3,593 genes). In addition, *O. massiliensis* and *O. iheyensis* shared a mean nucleotide sequence similarity of 80.49% at the genome level (range 70.03-98.32%).

### Prophage genome properties

Prophage Finder [59] was used to identify potential proviruses in *O. massiliensis* strain N’Diop^T^ genome. The genome contains at least one genetic element of around 48.9 kb (with a GC content of 38.9%), we named OM1, on contig 65. A total of 51 open reading frames (ORFs) were recovered from OM1, larger than 160 nucleotides and most of them (42) encode proteins sharing a high identity with proteins found in *Clostridiales* genus viruses. The preliminary annotation of OM1 was performed and the majority of the putative genes (31) encode hypothetical proteins. The ORFs with an attributed function (21) encode proteins involved in DNA packaging, cell lysis, tail structural components and assembly, head structural components and assembly, lysogeny control, DNA replication, recombination and modification. Fifty one of the ORFs are located on one strand and 1 on the opposite strand.

## Conclusion

Bacteria included in genera *Oceanobacillus* and *Ornithinibacillus* are closely related. The inclusion of these genera is based only on the iso-_C15:0_/anteiso-_C15:0_ ratio (>1 for the *Ornithinibacillus* genus and <1 for the *Oceanobacillus* genus) and the peptidoglycan type (A4β for the *Ornithinibacillus* genus and A1γ for the genus *Oceanobacillus* genus). All the other criteria proposed are not valid for all the representatives of the genus, or can be discordant when manipulations are performed in different laboratories.

On the basis of phenotypic, phylogenetic and genomic analyses, we propose the creation of *Oceanobacillus massiliensis* sp. nov. that contains the strain N’Diop^T^. This bacterium has been found in Senegal.

In the future, sequencing and comparison of several genomes of *Oceanobacillus* and *Ornithinibacillus* representatives will allow better understanding of these taxa.

### Description of *Oceanobacillus massiliensis* sp. nov.

***Oceanobacillus massiliensis*** (mas.si.li.en'sis. L. masc. adj. *massiliensis* of Massilia, the old Roman name for Marseille, where the type strain was isolated). Cells are aerobic, Gram-positive, straight, and motile rods. Catalase-positive and oxidase-positive. Growth occurred in aerobic conditions. Optimal growth occurs at 30-37 °C, pH 7-8 and NaCl range of 0,5-5%. After 24 hours growth on sheep blood agar, surface colonies are up to 2-5 mm in diameter, smooth, circular, greyish and shiny. The rods measure 1.2-1.9 μm in length and 0.4-0.7 μm in diameter (as determined by electron microscopy). Using Api ZYM, activities of esterase (C4), esterase lipase (C8) and α-glucosidase are detected. Activities of alkaline phosphatase, lipase (C14), leucine arylaminidase, valine arylaminidase, cystine arylaminidase, trypsin, α-chemotrypsin, acid phosphatase, naphthol-AS-BI-phosphohydrolase, β-galactosidase, β-glucuronidase, β- glucosidase, N-acetyl-β-glucosaminidase, α-mannosidase and α-fucosidase are not detected. Using Api 50CH strips after an incubation time of 48 h acid is produced from glycerol (weakly), D-xylose, D-glucose, D-fructose, D- mannose, D-mannitol, N- acetylglucosamine, esculin ferric citrate, salicin, D-cellobiose, D-maltose and D-trehalose. Acid is not produced from erythitol, D-arabinose, L-arabinose, D-ribose, L-xylose, D-adonitol, methyl-βD-xylopyranoside, D-galactose, L-sorbose, L-rhamnose, dulcitol, inositol, D-sorbitol, methyl-αD-mannopyranoside, methyl-αD-glucopyranoside, amygdalin, arbutin, D-lactose, D-melibiose, D-saccharose, inulin, D-melezitose, D-raffinose, amidon, glycogen, xylitol, gentiobiose, D-turanose, D-lyxose, D-tagatose, D-fucose, L-fucose, D-arabinol, L-arabinol, potassium gluconate, potassium 2-ketogluconate and potassium 5-ketogluconate. Using Api 20 NE, hydrolysis of esculine is positive. Nitrate reduction, indole production, glucose fermentation, arginine dihydrolase, urease, gelatin hydrolysis and β-galactosidase are negative.

The respiratory quinones are MK-7 (93%) and MK-6 (7%). The major fatty acids are C15:0 anteiso (63.24%) and C17:0 anteiso (26.86%). The G+C content of the genomic DNA is 40.35-40.8%.

The type strain, which was isolated from human feces, is N’diop^T^. It has been deposited in the Collection de Souches de l’Unité des Rickettsies, Marseille, France, as CSUR P132^T^ and in the Leibniz Institute DSMZ-German Collection of Microorganisms and Cell Cultures, as DSM 24644^T^.
